# A Novel Nonsense Mutation in the *MIP* Gene Linked to Congenital Posterior Polar Cataracts in a Chinese Family

**DOI:** 10.1371/journal.pone.0119296

**Published:** 2015-03-24

**Authors:** Zixun Song, Lianqing Wang, Yaping Liu, Wei Xiao

**Affiliations:** 1 Department of Ophthalmology, Shengjing Hospital of China Medical University, Shenyang, Liaoning, 110004, China; 2 Department of Medical Genetics, Institute of Basic Medical Sciences, Chinese Academy of Medical Sciences & Peking Union Medical College, Beijing 100005, P. R. China; University of Missouri-Columbia, UNITED STATES

## Abstract

**Purpose:**

To detect the causative mutation for congenital posterior polar cataracts in a five-generation Chinese family and further explore the potential pathogenesis of this disease.

**Methods:**

Coding exons, with flanking sequences of five candidate genes, were screened using direct DNA sequencing. The identified mutations were confirmed by restriction fragment length polymorphism (RFLP) analysis. A full-length wild-type or an Y219* mutant aquaporin0 (AQP0) fused with an N-terminal FLAG tag, was transfected into HEK293T cells. For co-localization studies, FLAG-WT-AQP0 and Myc-Y219*-AQP0 constructs were co-transfected. Quantitative real-time RT-PCR, western blotting and immunofluorescence studies were performed to determine protein expression levels and sub-cellular localization, respectively.

**Results:**

We identified a novel nonsense mutation in *MIP* (c.657 C>G; p.Y219*) (major intrinsic protein gene) that segregates with congenital posterior polar cataract in a Chinese family. This mutation altered a highly conserved tyrosine to a stop codon (Y219*) within AQP0.When FLAG-WT-AQP0 and FLAG-Y219*-AQP0 expression constructs were singly transfected into HEK 293T cells, mRNA expression showed no significant difference between the wild-type and the mutant, while Y219*-AQP0 protein expression was significantly lower than that of wild-type AQP0. Wild-type AQP0 predominantly localized to the plasma membrane, while the mutated protein was abundant within the cytoplasm of HEK293T cells. However, when FLAG-WT-AQP0 andMyc-MU-AQP0were co-expressed, both proteins showed high fluorescence in the cytoplasm.

**Conclusions:**

The novel nonsense mutation in the *MIP* gene (c.657 C>G) identified in a Chinese family may cause posterior polar cataracts. The dominant negative effect of the mutated protein on the wild-type protein interfered with the trafficking of wild-type protein to the cell membrane and both the mutant and wild-type protein were trapped in the cytoplasm. Consequently, both wild-type and mutant protein lost their function as a water channel on the cell membrane, and may result in a cataract phenotype. Our data also expands the spectrum of known *MIP* mutations.

## Background

Congenital cataracts are eye diseases characterized by an opacity of the lens, presenting at birth or shortly thereafter [1], and are responsible for the majority of visual impairment cases in children worldwide, despite improvements in surgical techniques in recent years [2]. Approximately 8.3–25% of congenital cataracts are inherited [3]. The nonsyndromic form of congenital cataract displays a mostly autosomal dominant inheritance pattern with complete penetrance [4].

Congenital cataractis a phenotypically and genetically heterogeneous disorder [4]. A number of such cataract phenotypes have been recognized, such as: whole lens, anterior polar, posterior polar, cortical, nuclear, lamellar, cerulean (or blue dot), coralliform, sutural, and pulverulent cataract [5, 6]. Although this disease shows highly variable expressivity, it is believed that there is a certain genotype-phenotype correlation to some extent [3]. More than 35 independent loci and 25 cataract-related genes have been identified that were associated with nonsyndromic autosomal dominant congenital cataract (ADCC) [7].

The *MIP* gene encodes a major intrinsic protein, also known as aquaporin0 (AQP0), which acts as a water channel [8]. Several studies have confirmed that aquaporins are functionalonly in the tetrameric form in the membrane [9–12] and thatonly aquaporinsin the form of tetramers can conduct water [13]. AQP0 is proposed to facilitate water removal [14] and it plays a critical role in controlling the water content of lens fiber cells [15]. AQP0 forms not only water pores, but also ‘thin lens junctions’ that AQP0 may also be involved in fiber-fiber adhesion [16]. AQP0 compacts highly ordered gamma-crystallins in lens fibers [17], and minimizes extracellular space to maintain an elevated refractive index for transparency.

## Methods

### 1 Study participants

**Fig 1:**
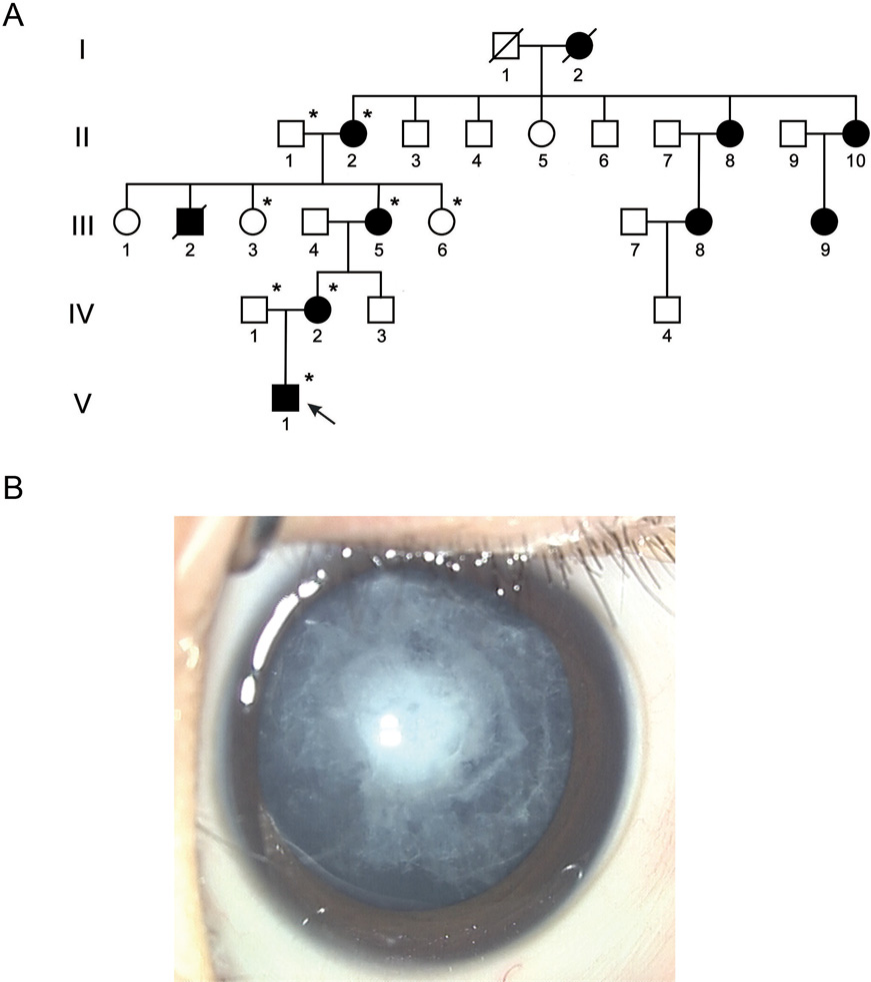
Clinical evaluation. **A: Pedigree of the five-generation Chinese family with autosomal dominant congenital cataract (ADCC).** Squares and circles indicate males and females, respectively. Filled symbols indicate affected members and empty symbols indicate unaffected individuals. The diagonal line indicates a deceased family member and the arrow indicates the proband. Family members whose DNA was analyzed by sequencing and restriction enzyme digestion are indicated by asterisks. **B: Photograph of the right eye of the proband.** The photograph (diffuse illumination) of the proband (V: 1) before surgery shows a posterior polar cataract with cotton-like opacities in the posterior subcapsular cortex. The same phenotype was noted bilaterally.

A five-generation family with autosomal dominant posterior polar cataract, living in the Liaoning province of China, was invited to participate in our study except the ones in first generation, who had passed at the time of examination (Fig. 1A). Each participant’s affected status was determined by their medical history or a detailed ocular examination, including visual acuity, anterior segment examination by slit lamp, and fundus examination with dilated pupils. A total of 200 unrelated participants, without a family history of congenital cataracts, were recruited as controls. Informed consent was obtained from all participants. This study adhered to the tenets of the Declaration of Helsinki and was approved by the Ethics Committee of Shengjing Hospital. Written informed consent was obtained from each patient, and we obtain written informed consent from the guardians on behalf of the children enrolled in our study.

### 2 Mutation screening

Blood samples were collected from both affected and unaffected family members as shown in Fig. 1A. Genomic DNA was extracted from peripheral blood samples using the QlAamp DNA Blood Mini Kit (Qiagen; Valencia, CA, USA).

Five functional candidate genes that are mostly involved in posterior polar ADCC were selected as determined from the Online Mendelian Inheritance in Man (OMIM) database. Polymerase chain reaction (PCR) was employed to amplify all coding exons and exon-intron flanking regions of the candidate genes: *CRYAA*, *CRYAB*, *PITX3*, *CHMP4B*, and *MIP*, using previously published primer sequences and cycling conditions [1, 18, 19]. PCR products obtained from the proband and one unaffected family member were sequenced.

### 3 Restriction fragment length polymorphism (RFLP) analysis

RFLP demonstrated that the c.657 C>G substitution in the *MIP* gene abolished an *Rsa*I restriction site. DNA sequences from all members of the family and 200 unrelated controls were amplified by PCR using the primers: 5’-TCTACAGGTGTACTGGGTAGG-3’ (forward) and 5’-GCCTGGGTGTTCAGTTCAACA-3’ (reverse). PCR product (10 μL) was digested with *Rsa*I(New England Biolabs; Ipswich, MA, USA) at 37°C for 30min and separated on a 2.5% agarose gel, along with a DL2,000 DNA Marker (TaKaRa; Dalian, China).

### 4 Human AQP0 cDNA and expression constructs

#### 4.1 Wild-type (WT) AQP0

The coding sequence of wild-type *MIP* (AQP0) was amplified by PCR from human kidney first-strand cDNA (Human multiple tissuescDNA panels; BD Biosciences; Palo Alto, CA, USA). The forward primer used in PCR contained a *Bam*HI site (5’-CGCTGGATCCATGTGGGAACTGCGATCAGC-3’), and reverse primer contained an *Xho*I site (5’-CCGACTCGAGCTACAGGGCCTGGGTGTTCA-3’).

#### 4.2 Mutant (Y219*) AQP0

To introduce the mutation (c.657 C>Gp.Y219*) into AQP0, site-directed mutagenesis was used with the following oligonucleotide primer and its complement: sense primer, 5’-GGGCAGCCTCCTGTAGGACTTTCTTCTCTTC-3’; antisense primer, 5’-GAAGAGAAGAAAGTCCTACAGGAGGCTGCCC-3’.

#### 4.3 Plasmid construction

To create FLAG-AQP0 and Myc-AQP0 fusion proteins, the PCR products of WT-AQP0 and Y219*-AQP0 were digested with *Bam*HI and *Xho*I restriction enzymes, respectively, purified with a PCR purification kit (Tiangen Biotech, Beijing, China), and subsequently cloned into the digested mammalian expression vector, pCMV-3Tag-6(Agilent Technologies, Shanghai, China), which contained an N-terminal triple FLAG epitope tag;and pCMV-3Tag-7 (Agilent Technologies, Shanghai, China), which contained an N-terminal tripleMyc epitope tag. All of the constructs were verified by direct sequencing.

### 5 Cell culture and transient transfection

Human embryonic kidney 293T (HEK 293T) cells were cultured in Dulbecco’s Modified Eagle’s Medium (DMEM) supplemented with 10% fetal bovine serum (FBS) in a humidified atmosphere containing 5% CO_2_ at 37°C. Wild-type (WT-AQP0), mutant AQP0 (Y219*-AQP0), and control plasmids (empty pCMV-3Tag-6 orpCMV-3Tag-7) with FLAG or Myc tag were transfected, respectively, or cotransfected into HEK 293T cells in comparable amounts using Lipofectamine 2000 (Invitrogen Corporation, Carlsbad, CA, USA).

### 6 Relative mRNA expression

For the MIP expression analysis, quantitative real-time RT-PCR was appliedto detect therelative mRNA expression of both wild-type and mutant. Total RNA was isolated from the HEK293T cells 24h after transfection. RNA samples were treated withRecombinant RNase-Free DNase (TaKaRa; Dalian, China). The first strands of cDNA were transcribed with GoScript Reverse TranscripionSysterm (Promega; Madison, USA) according to the manufacturer’s protocol. Quantitative PCR was carried out using SYBR Premix Ex Taq (TaKaRa; Dalian, China)withprimers for MIP(Forward: 5’- GGAAACCTAGCACTCAACACG-3’; Reverse: 5’- CTCGTCGTATGTGGCAAAGAT-3’) andβ-actin (Forward: 5’-CATGTACGTTGCTATCCAGGC-3’; Reverse: 5’-CTCCTTAATGTCACGCACGAT-3’). Reactions were run in the Rotor-Gene 6000 real-time rotary analyzer (Corbett Life Science) at 95°C for 6 min and then 40 cycles of 95°C for 10 s, 60°C for 15 s and 72°C for 20 s. All samples were analyzed in four replicates and normalized to median β-actin expression. The quantitative real-time RT-PCR experiments were repeated three times.

### 7 Proteinexpression

HEK 293T cells were harvested 24 h after transfection and analyzed by Western blotting for the expression of FLAG-WT-AQP0 and FLAG-Y219*-AQP0. HEK 293T cells were lysed in RIPA lysisbuffer and total cell extracts were separated by 15% SDS-PAGE gel electrophoresis, transferred to PVDF membranes and then incubated with mouse M2 monoclonal anti-Flag and anti-β-actin IgG (Sigma-Aldrich, Saint Louis, USA) at 1:1000 dilution in TBST buffer (10 mMTris, pH 7.5, 150 mMNaCl and 0.5% Tween-20) containing 5% nonfat dried milk at 4°Covernight, followed by HRP-conjugated goat anti-mouse IgG(1:3000; Pierce, Rockford, USA). Membranes were treated with enhanced chemiluminescence (ECL) reagents (Super Signal West Femto maximum sensitivity substrate, Thermo Fisher Scientific, Rockford, USA), followed by exposure to X-ray films. All samples were normalized relative to β-actin protein expression.

### 8 Subcellular localization

Wild-type and mutant AQP0 with FLAG tag were transfected separately into HEK 293T cells. For co-localization studies, FLAG-WT-AQP0 and Myc-Y219*-AQP0 constructs were co-transfected. Immunofluorescence studies were performed as described by Varadaraj et al [20]. Briefly, 24 h after transfection, cells cultured on glass coverslips were fixed in buffer containing 4% paraformaldehyde, and then counterstained with the nucleus-staining dye, DAPI. HEK 293T cells were subjected to immunofluorescence staining using c-Myc (9E 10) mouse monoclonal IgG (Santa Cruz Biotechnology, Shanghai, China) and rabbit anti-FLAG IgG(MEDICAL & BIOLOGICAL LABORATORIES CO.,LTD., Beijing, China), followed by Dylight 594 goat anti-mouse IgG and Dylight 488 goat anti-rabbitIgG(Earthox, San Francisco, CA,USA). All samples were analyzed by Olympus IX81 confocal fluorescence microscopy. Images were digitized and merged using FV1000 Viewer (Ver.3.0a) software (Olympus). Cells expressing the pCMV-3Tag-6or pCMV-3Tag-7 expression plasmid were used as negative controls. The assay was repeated three times.

## Results

### 1 Clinical evaluation

We identified a five-generation Chinese family with a clear diagnosis of posterior polar ADCC (Fig. 1A). The proband (V: 1) was a 3-month-old boy with a complaint of opacity in both lenses shortly after birth. He presented with bilateral posterior polar cataracts showing cotton-like opacities in the posterior subcapsular cortex (Fig. 1B). No other ocular or systemic abnormalities, or symptoms, were detected. The child underwent surgery shortly afterwards.

Several members of the child’s family showed similar congenital cataracts. Autosomal dominant inheritance was supported by the presence of affected individuals in each generation. The best corrected visual acuity of the affected patients ranged from 0.1 to 0.4, without complaint of decreased visual acuity.

### 2 Mutation analysis

**Fig 2:**
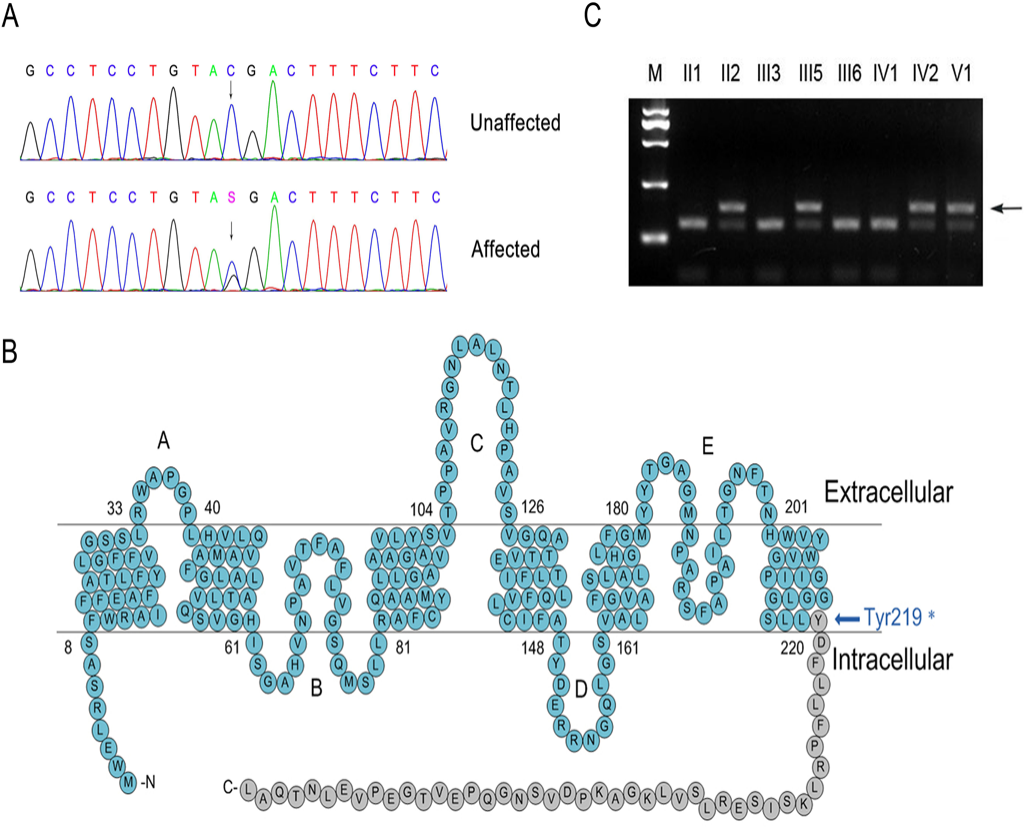
A novel nonsense mutation (c.657C > G; p.Y219*) in *MIP*/AQP0 in a Chinese family with posterior polar ADCC. **A: DNA sequences of *MIP* in unaffected and affected individuals.** The upper chromatogram of the DNA sequence from an unaffected individual (III: 3) shows only the wild-type AQP0 allele, which encodes tyrosine (TAC) at codon 219. The lower sequence chromatogram from the affected proband (V: 1) shows both C and G (S) at position 657 (arrow); thus, the mutant allele contained a C to G transition, which altered the Tyr to a stop codon (TAG).**B: A schematic diagram showing the presumed membrane topology of aquaporin0 (AQP0).** The depicted mutated portion (gray circles) illustrates the premature truncation of the protein. Amino acid residue 219 is located within the 6th transmembrane domain (blue arrow). **C: RFLP analysis shows the C>G transversion, which co-segregated with disease in a family.** The PCR product was 187bp in length and contained two *Rsa*I sites (GTAC). The unaffected allele yielded three fragments (12bp, 45bp, and 130bp) after *Rsa*I digestion, whereas the affected allele yielded four (12bp, 45bp, 130bp, and 175bp). Only the affected allele displayed the 175bp band (arrow). M indicates the DNA ladder.

By directly sequencing candidate genes, previously identified to cause nonsyndromic posterior polar ADCC, we identified a heterozygous nucleotide change, C > G, at position 657 (c.657C > G) of the *MIP* gene (Fig. 2A). Aquaporin0 (AQP0), also known as the lens’s major intrinsic protein, is encoded by the *MIP* gene (Fig. 2B). The nucleotide change altered a highly conserved tyrosine (TAC) to a stop codon (TAG) at the 219th amino acid position (p.Y219*) of AQP0.

RFLP analysis demonstrated that the c.657C > G substitution abolished an *Rsa*I restriction site confirming the mutation, and that the nucleotide substitution co-segregated with the disease phenotype (Fig. 2C). The c.657C > G substitution was present in all affected individuals who underwent DNA analysis (Fig. 2C), but was not observed in unaffected family members or in 200 unrelated controls (data not shown).

### 3 Expression levels of FLAG-WT-AQP0 and FLAG-Y219*-AQP0 in cultured cells

**Fig 3:**
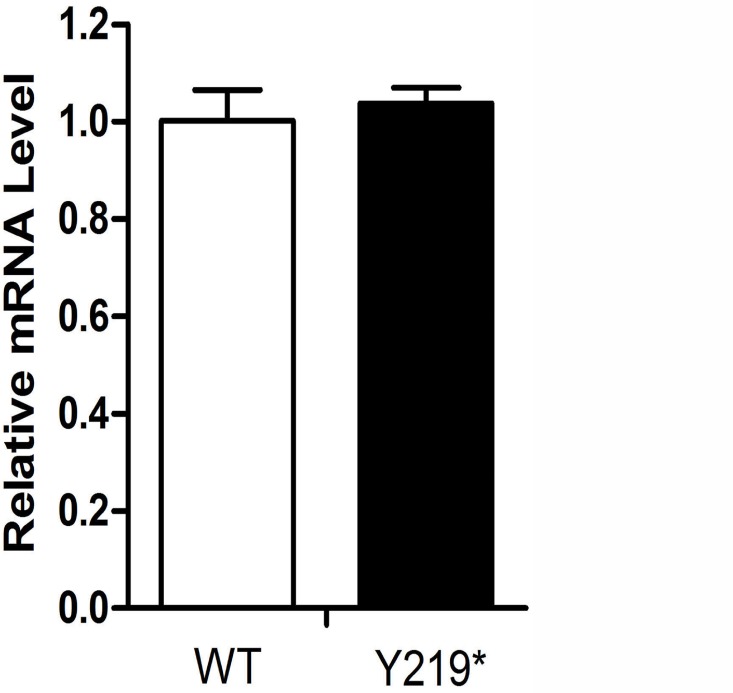
Quantitative analysis of *MIP* gene expression in HEK 293T cells. Cells transfected with wild-type (WT) or mutated (Y219*) AQP0 expression constructs show a similar relative *MIP* mRNA expression level. All samples were analyzed in four replicates and normalized to median β-actin expression.

There was no significant difference between wild-type and the mutant in mRNA level by quantitative real-time RT-PCR (Fig. 3).

**Fig 4:**
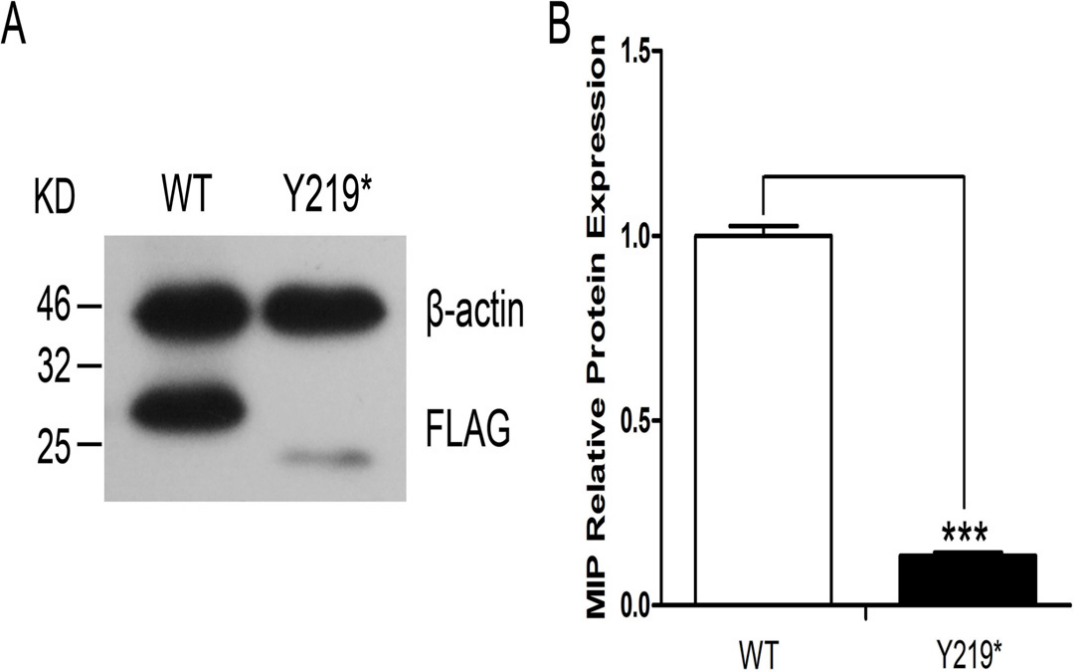
Protein expression levels of WT- and Y219*-AQP0 transfected into HEK 293T cells. Western blots were performed with the anti-FLAG as indicated.β-actin was used as the loading control. Cells with the mutated (Y219*) AQP0 construct showed an 87% reduction in AQP0 protein level compared to cells with wild-type AQP0. ***P<0.01

The MIP gene encodes the major intrinsic protein, also known as aquaporin0 (AQP0). AQP0 is inserted into the plasma membrane and acts as a water channel. Wild-type WT-AQP0 or mutant Y219*-AQP0 proteins with FLAG tags were expressed in HEK 293T cells. Protein expression levels were assessed by immunoblot analysis of total cell extracts using anti-FLAG. In cells transfected with FLAG-WT-AQP0, a 30 kDa band was detected, which corresponded to the size of the full length AQP0 plus the Flag protein (Fig. 4). A band was also detected in cells transfected with FLAG-Y219*-AQP0, but was less abundant and demonstrated slightly faster electrophoretic mobility compared to the wild-type protein, indicating a less expressed truncated protein (Fig. 4). Expression levels of the β-actin loading control in both cell extracts were comparable. These results indicate that in transfected cells, the Y219* mutation reduced protein levels of the AQP0, which appeared to have lower MW.

### 4 Subcellular localization of WT- AQP0 and Y219*-AQP0

**Fig 5:**
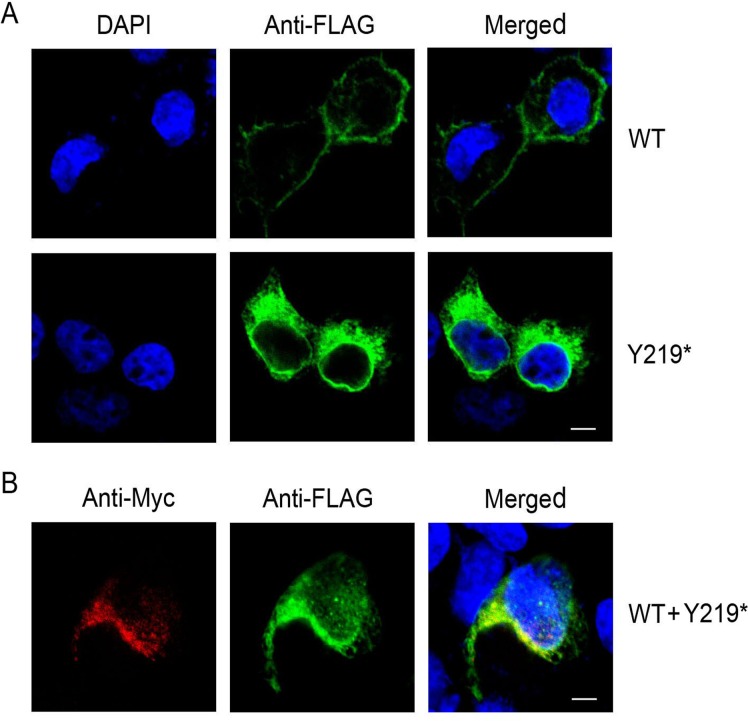
Subcellular localization of WT-AQP0 and Y219*-AQP0in HEK 293T cells, 24 h after transient transfection. Bar 5μm. A: Localization of singly transfected FLAG-tagged wild-type (WT) and mutated (219*) AQP0 proteins. Photomicrographs show the distribution of immunoreactive FLAG-tagged AQP0 (green) and DAPI-stained nuclei (blue) B: Localization of co-transfected FLAG-WT-AQP0 and Myc-219*-AQP0 proteins. Photomicrographs show the distribution of immunoreactiveMyc-tagged 219*-AQP0 (red), FLAG-tagged WT-AQP0 (green) and DAPI-stained nuclei (blue)

The subcellular localization of wild-type FLAG-WT-AQP0 or mutated FLAG-Y219*-AQP0 in transfected HEK 293T cells was determined by confocal fluorescence microscopy (Fig. 5A). As expected, wild-type AQP0 was predominantly localized in the plasma membrane, while the mutated AQP0 was localized abundantly in the cytoplasm of HEK 293T cells. Green fluorescence was not detected in HEK 293T cells transfected with empty vector pCMV-3Tag-6 (data not shown). These images suggest that the truncated protein (mutated AQP0), with a deletion of its C-terminus, impaired the trafficking of the expressed mutant protein to the plasma membrane.

Moreover, when FLAG-WT-AQP0 andMyc-MU-AQP0were co-expressed, both proteins showed high flurescencein the cytoplasm (Fig. 5B). The merged image of FLAG-WT-AQP0 and Myc-MU-AQP0 taken from the same cell showed co-localization of the wild-type and mutant proteins. Therefore, not only the mutant but the wild-type failed to properly traffic to the plasma membrane.

## Discussion

In this study, we identified a novel nonsense mutation (c.657C > G; p.Y219*) in *MIP*/AQP0 in a Chinese family with posterior polar ADCC. Our study supports the hypothesis that this mutation causes the posterior polar ADCC phenotype in this family.

AQP0 is a member of the aquaporin family of proteins that function as water channels and adhesion molecules within eye lens cells. In 2000, Berryet et al [21] identified the first mutation affecting the AQP0/*MIP* gene. To date, 14 mutations in AQP0/*MIP* have been identified, including: nine missense mutations [1, 21–29]; two splicing mutations [30, 31]; and one small deletion [32]. These mutations mainly caused nuclear, lamellar, sutural and posterior polar cataract. AQP0 is the most abundant membrane protein in the posterior pole and nuclear fibers of the lens. This could well explain why the opacity caused by mutations in *MIP* is mainly present in the nucleus and posterior of the lens.

In the present study, we examined the localization, dominant negative effect of the mutant protein produced by c. 657C > G, p.Y219* mutation in the *MIP* gene. Our results suggest that the c. 657C > G, p.Y219* mutation has several consequences that may contribute to the pathogenesis of ADCC in the family studied.

When FLAG-WT-AQP0 and FLAG-Y219*-AQP0 expression constructs were singly transfected into HEK 293T cells, we observed that mRNA expression showed no significant difference between the wild-type and the mutant, while Y219*-AQP0 protein expression was significantly lower than that of wild-type AQP0,similar to that found for the G165D mutation [25]. On the other hand, the immunofluorescence result of truncated mutant protein indicated that there really were cells that showed considerable expression as shown in Fig. 5A. These results implied that the truncated protein (mutated AQP0) was made and then rapidly degraded.

However, the phenotype cannot be only attributed to the decrease in the expression level of the protein. When FLAG-WT-AQP0 and Myc-Y219*-AQP0 expression constructs were cotransfected into HEK 293T cells, not only the mutant but the wild-type AQP0 were trapped in cytoplasm and failed to reach its target-cell membranes—to perform the function. Phosphorylation of AQP0 at serine235 and 245, serving as a sorting signal [33, 34], was lost in the truncated mutant protein, which explains our observation that the mutant protein was retained in the cytoplasm. Altogether, trafficking problems had also previously been observed for G165D and G213Vfs*46 mutated AQP0, which were both retained in the endoplasmic reticulum (ER) [25, 32]. Aquaporins are synthesized as monomers, folded and assembled as a pack of AQP0 tetramers in the ER before transported and inserted into the plasma membrane [35, 36]. Kumar et al [20] suggested that the retention of the aggregation of conformationally unstable proteins in the ER may trigger ER stress. Our data indicated that both WT-AQP0 andY219*-AQP0 were trapped in the cytoplasm and also the level of Y219*-AQP0 protein was significantly decreased. These observations might be supportive evidence for the notion above suggested by Kumar et al [20].

AQP0 is a major permeability pathway for water in the lens [37]. As the lens is an avascular tissue, it relies heavily on a unique transport system to maintain its high protein concentration and low water content to keep it transparent and homeostatic [38]. We therefore postulated that the trafficking problem caused by Y219* mutation would severely decrease the number of available water channels in the plasma membrane and subsequently affect its water permeability. This may prevent the lens from maintaining the appropriate water content for homeostasis and decrease the transparency of hydrophobic lens fiber cells.

Besides of acting as water channel, AQP0 plays a major role as an adhesion molecule in the stacking of lens fiber cells. The lack of this function causes loss of stacking order and thus a turbidity of the lens, which is cataract [39].

In summary, we have described a novel nonsense mutation in *MIP* causing autosomal dominant congenital cataracts in a Chinese family. We showed that a Y219* mutation in AQP0 resulted in the loss of the entire intracellular C-terminus of AQP0 and the formation of a premature truncated protein. Both the mutant and wild-type protein were trapped in the cytoplasm. Consequently, both wild-type and mutant protein lost their function as a water channel on the cell membrane, and may result in a cataract phenotype. Our data also expands the spectrum of known *MIP* mutations.
